# Artificial Intelligence (AI) and Robotics in Elderly Healthcare: Enabling Independence and Quality of Life

**DOI:** 10.7759/cureus.42905

**Published:** 2023-08-03

**Authors:** Srikanta Padhan, Avilash Mohapatra, Senthil Kumar Ramasamy, Sanjana Agrawal

**Affiliations:** 1 Community and Family Medicine, All India Institute of Medical Sciences, Raipur, Raipur, IND; 2 Physiotherapy, All India Institute of Medical Sciences, New Delhi, IND; 3 Epidemiology and Public Health, State Health Resource Center, Raipur, IND; 4 Epidemiology and Public Health, All India Institute of Medical Sciences, Raipur, Raipur, IND

**Keywords:** geriatric rehabilitation, social robots, artificial intelligence, geriatric health, elderly population

## Abstract

We are being urged to redefine aging and only use positive terminology when discussing it. It is unacceptable to use a derogatory term like "aging tsunami." This is unfortunate because it comes at a time when geriatrics is precariously balancing itself. Geriatricians are growing far too slowly to supply an adequate number of medical professionals to meet the needs of the rapidly aging senior population. The global aging population poses significant challenges for healthcare systems and providing elderly care. In recent years, artificial intelligence (AI) and robotics have emerged as promising technologies to address these challenges by enabling independence and enhancing the quality of life for older adults. This review article examines the applications of AI and robotics in elderly care, focusing on their role in promoting independence, monitoring health, helping, and enhancing social interaction. The article also discusses the ethical considerations, challenges, and future directions in implementing AI and robotics in elderly care.

## Editorial

Population aging is a global issue; 703 million people aged 65 and up are expected to rise to 1.5 billion by 2050 [1]. One in six persons worldwide will be over 65 by 2050, based on the most recent population projections and estimates of the United Nations Population Division Department of Economic and Social Affairs (DESA) [2]. Ensuring older people can fully engage in all facets of communal life is a pressing requirement. However, older persons are often subjected to discrimination, neglect, agism, exclusion, and other violations [3]. It presents a growing need for effective elderly care solutions that can provide adequate support and maintain the independence and well-being of older adults. Artificial intelligence (AI) and robotics offer innovative approaches to address these challenges as technology advances. These technologies are revolutionizing many areas, including public health, medicine, physiotherapy, and other allied health services for older people, where they can help predict health risks and events, enable drug development, and support individualization of treatments [4]. The COVID-19 pandemic has placed a heavy burden on older people, especially those in long-term care facilities, reinforcing the demand for new strategies to help older adults. In this article, we tried to explore the importance of AI and robotics in improving the independence and quality of life among the elderly population.

AI in elderly care

Promoting Independence

AI-powered systems can assist older adults in performing daily activities, such as medication management, fall detection, and navigation, enabling them to live independently for longer [5]. Innovative home technologies with AI algorithms can detect deviations from standard behavior patterns and provide timely emergency alerts. In the context of aging, AI may also be used to support a more sophisticated level of decision-making in the home by older adults who are living independently or desire to do so. This includes the use of AI to automate home safety risk prevention and the capability to respond to emergencies in real time. The system can send a real-time alarm to the family, care facility, or medical agent without human assistance if it determines that something odd might occur (broadly) or something is wrong with the user's health practices or medical recommendations. In addition, AI-driven wearable devices can monitor vital signs and activity levels, promoting a healthier and more independent lifestyle.

Geriatric Population and Mental Health

Recent studies have shown that loneliness is a significant issue for older adults, contributing to cognitive impairments, depression, and frailty [6]. To help the lonely elderly, there is a need to create "Circle of Friends" programs adopting AI. In addition, there is a need for programs to lessen caretaker discomfort and for a greater understanding of carer stress employing AI.

Monitoring of Chronic Diseases

AI algorithms have the potential to revolutionize health monitoring for older adults. By analyzing data from wearable devices, electronic health records, and other sources, AI can provide real-time data analysis, detect early warning signs of diseases, and provide personalized treatment plans and recommendations. AI-enabled telemedicine platforms also enable remote monitoring and virtual consultations, improving access to healthcare for older adults in remote or underserved areas [7]. In addition to a diagnostic and management algorithm, humankind has created an iPad software with Reshma Merchant in Singapore for geriatric syndromes (RGA). It has been demonstrated that AI can read retinal scans like doctors [8]. AI will also be crucial in the deprescription process. These methods will be used often in medicine over the next 10 years. Other possible applications include the ongoing development of virtual medicine and improved assessment of osteoporosis and fracture risk concerning age, frailty, and life expectancy.

Robotics in elderly care

Assistive Robots

Robotic systems equipped with sensors and actuators can provide physical assistance to older adults with mobility support, personal hygiene, and household chores. These robots can be programmed to adapt to individual needs, providing personalized and responsive care [9]. Robotic exoskeletons and mobility aids enable older adults with mobility impairments to regain independence and perform activities they would otherwise struggle with. Robots with a mind are being created to help elderly patients in hospitals with their therapy. By physically touching humans, these robots can affect their emotional, physical, and social well-being. With this addition, older adults' spirits were seen to improve.

Social Robots

Social interaction is vital in combating social isolation and promoting mental well-being among older adults. Social robots offer companionship and engagement, providing emotional support and cognitive stimulation. These robots can engage in conversations, play games, and even assist in reminiscence therapy, improving older adults' overall quality of life. The robot's acceptance among older people is greatly influenced by its physical appearance. When dementia-stricken seniors were given companion animal robots, positive outcomes were discovered. Studies reveal that companion animal robots of the right size, weight, and shape can stimulate the brains of older people with dementia.

Geriatric rehabilitation and AI

Robots, exoskeletons, smart homes, wearable, voice-activated technology, and virtual reality applications are all AI technologies. One or more of these strategies might be very helpful regarding rehabilitation, offering emotional, practical, or material assistance and encouraging social and interpersonal interactions. A method for applying AI as a nursing care-support tool is based on its capacity to create robotic technology, which would lessen the labor of nursing staff in long-term care facilities. Aside from providing more alternatives for movement and living space, it is also predicted to offer psychological advantages [10].

Ethical considerations and challenges

**Figure 1:**
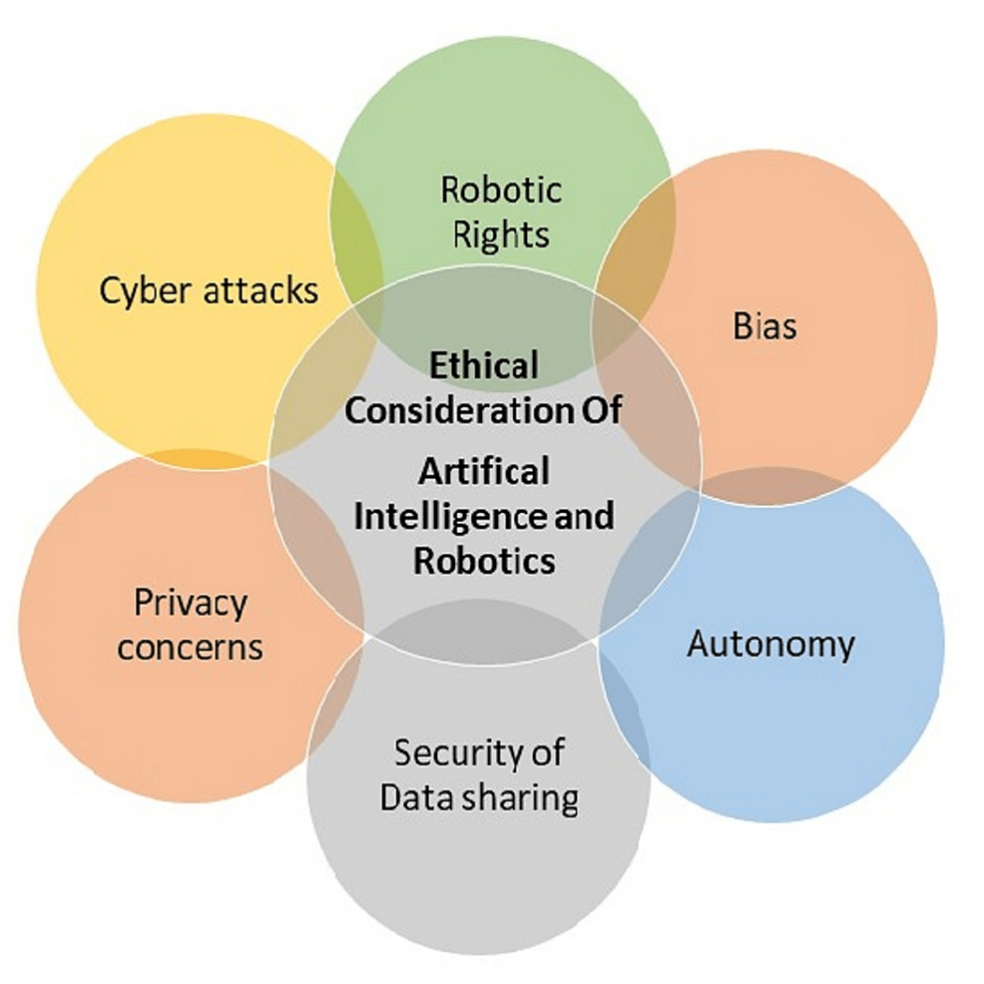
Ethical consideration of artificial intelligence and robotics

The integration of AI and robotics in elderly care raises critical ethical considerations. Privacy concerns regarding collecting and using personal health data must be addressed through robust security measures and clear consent procedures [11]. However, cloud technologies that allow for real-time data analytics from numerous sources across integrated organizations and data sharing need to be carefully evaluated, information security needs to be maintained, and the threat of cyberattacks needs to be minimized. In addition, the issue of autonomy arises when older adults rely heavily on AI and robotic systems, raising questions about decision-making and the potential loss of human connection. Developing guidelines and regulations that ensure these technologies' responsible and ethical use is crucial. The employment of robots by older persons may increase the danger of dishonesty, infantilization, and confusion among them as to why their human caretakers are using technology rather than face-to-face consultations (Figure 1).

Future directions and conclusion

The escalating global challenge of population aging necessitates urgent and innovative solutions to support older adults' independence and well-being. AI and robotics offer promising technologies to address the healthcare needs of the rapidly aging senior population. This review explored various applications of AI and robotics in elderly care, emphasizing their pivotal roles in promoting independence, monitoring health, facilitating social interaction, and aiding in geriatric rehabilitation. AI-powered systems enable personalized assistance, allowing older adults to live autonomously for extended periods. Innovative home technologies with AI algorithms swiftly detect emergencies and deviations in behavior patterns, ensuring prompt responses and heightened safety. AI-driven wearable devices monitor vital signs, foster healthier lifestyles among older people, and predict health risks, offering individualized treatment plans through telemedicine platforms. Integrating robotics complements AI, providing invaluable physical assistance and companionship, effectively combating social isolation, and enhancing mental well-being among older adults. However, ethical considerations are essential in implementing AI and robotics in elderly care, ensuring privacy and data security, and preserving human connection and autonomy. By thoughtfully regulating and embracing these technologies, we can empower older adults to lead fulfilling lives and improve the quality of elderly care globally.
